# Decreased testosterone secretion index and free testosterone level with multiple symptoms for late-onset hypogonadism identification: a nationwide multicenter study with 5980 aging males in China

**DOI:** 10.18632/aging.202227

**Published:** 2020-11-21

**Authors:** Honggang Li, Yiqun Gu, Xuejun Shang, Yuanzhong Zhou, Huiping Zhang, Liandong Zuo, Guangan Mei, Wei Xia, Huangtao Guan, Wenpei Xiang, Shanjie Zhou, Changchun Wan, Baojin Hao, Xubo Shen, Linguo Tang, Weixiong Wu, Ying Qi, Na Yu, Xiangbin Kong, Yaoping Chen, Yihong Yang, Xingrong Qing, Chengliang Xiong

**Affiliations:** 1 Institute of Reproductive Health/Center of Reproductive Medicine, Tongji Medical College, Huazhong University of Science and Technology, Wuhan 430030, China; 2 Wuhan Tongji Reproductive Medicine Hospital, Wuhan 430013, China; 3 National Health and Family Planning Key Laboratory of Male Reproductive Health, National Research Institute for Family Planning, Beijing 10081, China; 4 Department of Andrology, Jinling Hospital, School of Medicine, Nanjing University, Nanjing 210002, China; 5 School of Public Health, Zunyi Medical University, Zunyi 563006, China; 6 Guangzhou Women and Children’s Medical Center, Guangzhou 510180, China; 7 Technical Guidance Institute of Shanxi Province Family Planning Commission, Xi’an 710000, China; 8 Reproductive Medicine Centre, Peking University International Hospital, Beijing 102206, China; 9 People’s Hospital of Jinhu, Jinhu 211600, China; 10 School of Public Health, Guangdong Medical University, Guangzhou 524023, China; 11 Reproductive Medical Centre, Department of Obstetrics and Gynecology, Key Laboratory of Birth Defects and Related Diseases of Women and Children, Ministry of Education, West China Second University Hospital, Sichuan University, Chengdu 610041, China

**Keywords:** late-onset hypogonadism, aging, aging male, testosterone, testosterone secretion index

## Abstract

Late-onset hypogonadism (LOH) is a syndrome in middle-aged and elderly men caused by age-related testosterone deficiency. Age-related change of total testosterone (TT) of Asian males is different from Caucasian population, suggesting difference for LOH identification in Asians. A nationwide cross-sectional study involving six centers in China was conducted. Totally 6296 men aged 40-79 were recruited. After exclusions 5980 men were left for analyses. The serum TT level, was neither decreased with aging nor correlated with most hypogonadal symptoms. Instead, ten hypogonadal symptoms were found to be significantly correlated with free testosterone and testosterone secretion index, thus were chosen to form a concise scale. Further analysis identified a level of free testosterone <210 pmol/L, testosterone secretion index <1.8, and the concise scale score ≧17 could be diagnosed as having significantly aggravated LOH. This study developed an evidence-based criteria for LOH identification in Chinese population and may be adopted in other Asians. It includes the impaired testosterone secretion ability and deficiency of bioavailable testosterone, which should be the main cause in LOH pathogenesis despite normal TT levels, as well as correlated multiple hypogonadal symptoms. Our results may guide the LOH treatment to increase testicular function of testosterone secretion and bioavailable testosterone.

## INTRODUCTION

Late-onset hypogonadism (LOH) is defined as a clinical and biochemical syndrome associated with advancing age and is characterized by symptoms along with deficiency in serum testosterone levels [1]. Testosterone deficiency can adversely affect multiple organ functions and cause both sexual and nonsexual symptoms. The sexual symptoms of LOH include impotence or erectile dysfunction, low libido, and diminished frequency of morning erections, while the nonsexual symptoms include fatigue, depression, and decreased sense of vitality, among others. Because LOH adversely affects the quality of life in aging males with high prevalence (2%~40%) [2, 3]. its clinical significance is becoming increasingly recognized as many countries are facing an aging society. Specifically in Asia, which accounts for more than 60% of the world population, there are more than 8 hundred million aging men (>=40 years old) and the number is increasing (http://populationpyramid.net/).

Although LOH has been increasingly recognized as an age-related disorder, most men with LOH remain undiagnosed [1]. LOH diagnostic criteria have not been established for Asian men because there is no sufficient data to base on. For Caucasian men, a previous study suggested the presence of three sexual symptoms associated with a total testosterone (TT) level of less than 11 nmol/L and a calculated free testosterone (cFT) level of less than 220 pmol/L as the minimum criteria to identify LOH [2]. However, the serum concentration of TT, which shows age-associated decline in Caucasian men and is used as the primary biochemical parameter used to define testosterone deficiency for LOH identification [2, 4], do not decline or even increase with aging in Asian men [5–8]. Thus it is inappropriate to directly apply the criterion developed based on Caucasian population. LOH identification and its epidemiology are likely different among different ethnic groups. In fact, for many diseases, substantial racial or ethnic differences exist in many aspects, including the diagnostic criteria and prevalence [9, 10].

Based on the definition and pathophysiology of LOH [1]. LOH belongs to the organic and primary hypogonadism resulted from the failure of testis to produce physiological concentrations of testosterone [11, 12]. It is hypothesized that the ability of testicular testosterone secretion should be impaired resulting in testosterone deficiency in some, if not all, aging males, which in turn may cause LOH symptoms. The present study has carried out a nationwide, multi-center study in China aimed at establishing an evidence-based and comprehensive criteria for LOH diagnosis with the revelation of pathogenesis of LOH.

## RESULTS

### Participants

Totally 6296 men aged 40 to 79 years old (Supplementary Figure 1) from six representative areas of China attended this study. Totally 316 participants aged 40 to 79 years old were excluded from the analysis, with 217 because of incomplete response to questionnaires, and 99 because of known diseases of hypothalamus-pituitary-testis axis, or conditions may induce testicular impairment, or current use of medications may affect testosterone level. These exclusions left total 5980 men aged 40 to 79 for analyses. Men aged 22-39 (n=598) were recruited to obtain the baseline of hormones and response to questionnaires. All men (n=598) aged 22-39 completed the survey and were included in the analyses.

### Age trends of serum total testosterone

**Figure 1:**
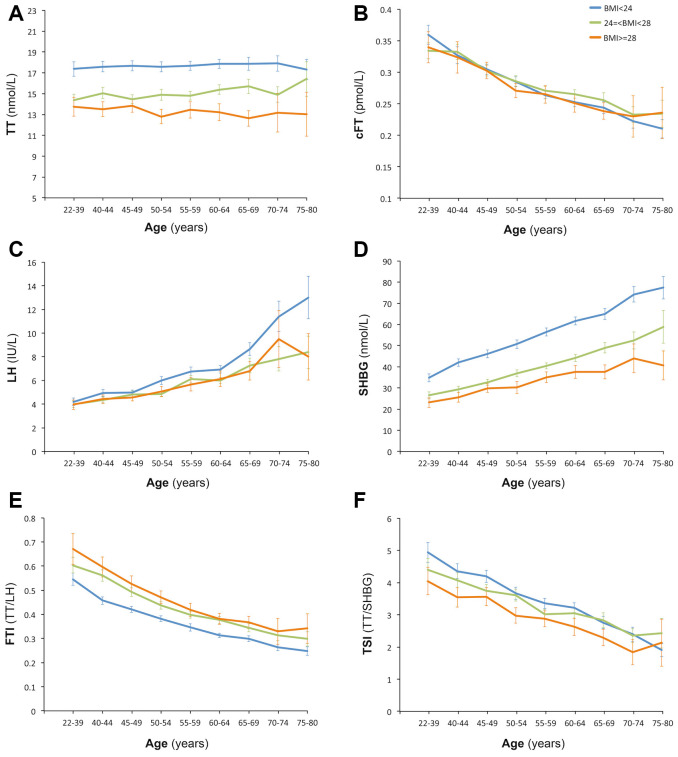
**Age trend of hormones.** Participants of each age was stratified according to the BMI standard for Chinese population: less than 24 kg/m^2^ (normal), 24 or greater to less than 28 kg/m^2^ (overweight), and ≥28 kg/m^2^ (obese). Pearson Correlation Analysis indicated that age was positively correlated with TT (**A**) (r=0.05, P<0.01), SHBG (**D**) (r=0.400, P<0.01) and LH (**C**) (r=0.360, P<0.01), and was negatively correlated with cFT (**B**) (r=-0.327, P<0.01), FTI (**E**) (r=-0.421, P<0.01) and TSI (**F**) (r=-0.295, P<0.01).

**Figure 2:**
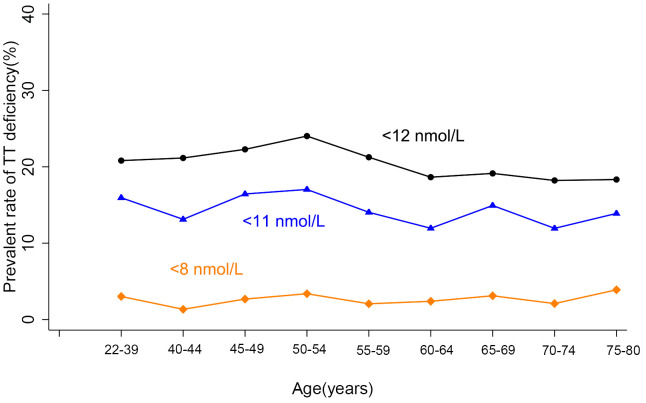
**Age trend of TT deficiency.** TT deficiency was defined with three kinds of standards: TT<8 nmol/L is the standard for TT supplement; <11 nmol/L and <12 nmol/L is the standard for diagnosis of LOH in EMAS study and other studies, respectively. No age trend of the occurrence of TT deficiency defined by any standard was observed.

Age trends of hormones of all participants were analyzed with three categories of BMI according to the standards for Chinese population: less than 24 kg/m^2^ (normal), 24 or greater to less than 28 kg/m^2^ (overweight), and ≥28 kg/m^2^ (obese) [13]. Serum TT showed significant difference among the three BMI categories (Supplementary Table 1) and a little fluctuation with aging, whereas no age trend was observed (Figure 1A). Considering some factors may influence TT and conceal the age trend of TT level. Potential factors associated with TT levels were further screened using stepwise multiple linear regression. BMI, residence, smoking, alcohol-drinking, and marital status, were found to be correlated with TT levels, whereas age was not after removing effects of these listed factors (Supplementary Table 2) in our population. We further analyzed the occurrence of several proposed abnormal TT levels (below 8, 11, and 12nmol/L, respectively) with aging [1, 2], and no age trend was observed (Figure 2).

### Age trends of hormones related with testosterone secretion and function

We then analyzed the age trends of other hormones and indexes related with testosterone secretion and function. Obvious negative age trend was observed in the cFT, which represents the bioavailable testosterone [14], and positive age trend was observed in luteinizing hormone (LH) and sex hormone-binding globulin (SHBG) (Figure 1B–1D). LH level was similar among the three groups before age 50, whereas it was slightly higher in the nonobese men between age 50 to 70, and significantly higher in the oldest nonobese men. SHBG level showed significant difference among the three groups and the differences widened with aging. Free testosterone index (FTI), the simplest calculation of bioavailable T, was significant different among the three groups and showed a negative age trend (Figure 1E). Testosterone secretion index (TSI), the ratio of TT to LH, which represents the secretion ability of testosterone, also showed a negative age trend, with significant difference between obese men and nonobese men (Figure 1F).

### Symptoms correlated with testosterone deficiency

Men aged 40-79 were randomly subdivided into a training set (n=2978) and a validation set (n=3002) (Supplementary Table 3) for further analysis. To identify symptoms associated with serum testosterone levels and secretion, all questions were screened with proportional ordinal logistic regression (Supplementary Table 4) in the training set. Candidate items were further confirmed by Mann-Whitney U test (Table 1). Consistent with age trends of hormones observed in our aging population, most (22 out of 24) of these items, including the three sexual symptoms included in the diagnostic criteria based on study of European population [2], were not correlated with TT levels, but most of them are correlated with cFT and FTI (21 items), or TSI (17 items). The 21 items correlated with cFT included 10 items from the aging males’ symptoms (AMS) scale [15, 16], 9 from the Medical Outcomes Study 36-Item Short-Form Healthy Survey, and 2 from the Beck Depression Inventory. However, most of the 11 items from the Medical Outcomes Study 36-Item Short-Form Healthy Survey and Beck Depression Inventory were similar to the 10 items from AMS (Table 1), which include 3 items from psychological subscale, 3 items from somatic subscale, and 4 items from sexual subscale. Moreover, 8 of the 10 items were also correlated with TSI. Thus these 10 items from AMS correlated with cFT were chosen to represent symptoms of testosterone deficiency, and to form the concise scale of AMS (cAMS).

**Table 1:** Correlations of symptoms with testosterone.

**Scale**	**Symptoms**	**Symptom^a^ prevalence**	***P* value**			
		%	TT	cFT	FTI	TSI
AMS						
	Increased need for sleep, often feeling tired	39.3	0.408	0.019	0.000	0.024
	Irritability	35.2	0.002	0.001	0.000	0.160
	Nervousness	26.3	0.041	0.006	0.000	0.828
	Physical exhaustion / lacking vitality	53.7	0.877	0.028	0.014	0.005
	Decrease in muscular strength	51.9	0.971	0.000	0.000	0.001
	Feeling burnt out, having hit rock-bottom	33.7	0.934	0.010	0.003	0.008
	Decrease in beard growth	22.2	0.511	0.000	0.000	0.000
	Decrease in ability/frequency to perform sexually	67.8	0.320	0.019	0.000	0.000
	Decrease in the number of morning erections	66.1	0.086	0.004	0.000	0.000
	Decrease in sexual desire/libido	65.7	0.272	0.003	0.000	0.000
SF-36						
	Compared with one year ago, how would you rate your health in general now?	42.8	0.670	0.000	0.000	0.365
	Does your health now limit you in moderate activities?	29.1	0.003	0.000	0.001	0.008
	Does your health now limit you in climbing several flights of stairs	32.1	0.030	0.001	0.042	0.000
	Does your health now limit you in climbing one flight of stairs	14.7	0.551	0.000	0.000	0.007
	During the past 4 weeks cut down the amount of time spent on work or other activities	27.2	0.528	0.000	0.000	0.000
	During the past 4 weeks accomplished less than you would like	29.4	0.977	0.000	0.000	0.000
	During the past 4 weeks were limited in the kind of work or other activities	29.6	0.613	0.000	0.000	0.000
	During the past 4 weeks had difficulty performing the work or other activities	31.4	0.629	0.002	0.000	0.000
	During the past 4 weeks have you been a very nervous person?	72.8	0.216	0.725	0.231	0.179
	During the past 4 weeks did you have a lot of energy?	98.3	0.685	0.439	0.476	0.379
	During the past 4 weeks, how much of the time has your physical health or emotional problems interfered with your social activities?	55.6	0.082	0.039	0.009	0.003
Beck Depression Inventory						
	Past failure	15.6	0.885	0.001	0.000	0.197
	Lost of interest	15.8	0.711	0.304	0.746	0.380
	Lost of energy	22.7	0.071	0.000	0.000	0.001

^a^Symptomatic man was defined as has any grade of symptom or answer yes to symptoms.

cAMS score was then calculated, and different cut-off values were set to divide the training set into two groups. Student t test was used to compare cFT and TSI levels between the two groups. When the cAMS score reached 17, significant difference in both cFT and TSI between the two groups divided first appeared (Supplementary Table 5). Given that symptoms of LOH were caused by the deficiency in testosterone, including secretion and functional testosterone, we determined the cAMS score of 17 to be the cut-off value for the screening symptoms of LOH.

### Thresholds of cFT and TSI for LOH identification

**Figure 3:**
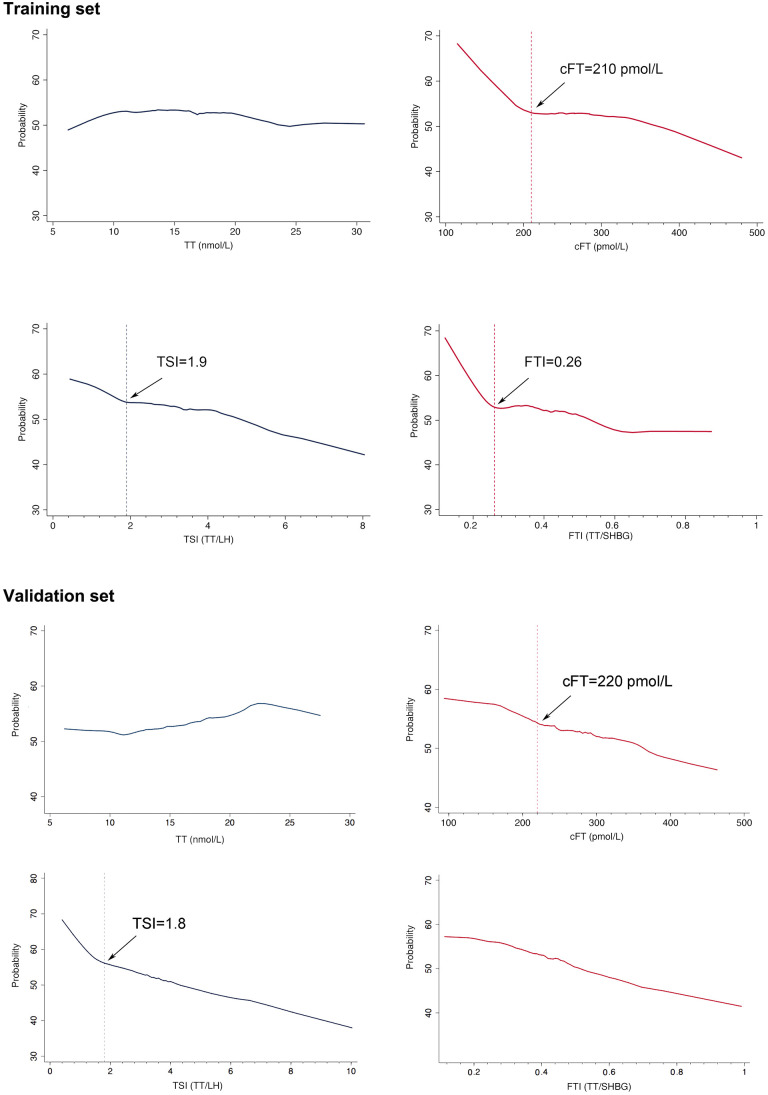
**Probability of symptom (cAMS>=17) on the basis of levels of bioavailable testosterone (cFT) and testosterone secreting index (TSI).** Symptom was defined by cAMS score (>=17), which contained 10 items related with cFT. Obvious increases of the trend of probability of symptom were observed with decreased cFT, and TSI. Thresholds for the significant changes of the probability of symptom with hormones were denoted by vertical lines.

The cAMS score of 17 was used to classify aging men in the training set into symptomatic and asymptomatic subjects. The trend of the probability of symptom along with the change of cFT, FTI, TSI, TT was further analyzed by Lowess smoothing. Increases of the probability of symptom were observed with decreased cFT, FTI, and TSI, but not with TT (Figure 3). In the training set, thresholds for the significant increase of the probability of symptom were approximately 210 pmol/L for cFT, 0.26 for FTI, and 1.9 for TSI, while no threshold was found for TT. In the validation set, similar results were obtained, with thresholds of 220 pmol/L for cFT, and 1.8 for TSI. The thresholds of cFT and FTI were further identified by piecewise repression in both the training set and the validation set. Consistent results were obtained for cFT<210 pmol/L and TSI<1.8 in these two sets (Table 2).

**Table 2:** Identification of cFT and TSI thresholds.

**Symptoms**	**Linear splines**	**OR (95% CI)**	
		**Training set**	**Validation set**
cAMS>=17	**cFT<210 pmol/L**	**42.10 (2.29, 765.10)**	**172.43 (5.75, 5218.68)**
	cFT>=210 pmol/L	9.39 (6.41*10^-7^, 1.38*10^8^)	6.36 (1.44*10^-6^, 2.81*10^7^)
	cFT<220 pmol/L	5.64 (3.05*10^-6^, 1.03*10^7^)	105.64 (2.92, 3827.63)
	cFT>=220 pmol/L	44.26 (2.20, 880.07)	6.17 (1.11*10^-5^, 3.47*10^6^)
	**TSI<1.8**	1.24*10^5^ (1.04*10^5^, 1.45*10^5^)	1.21*10^5^ (1.01*10^5^, 1.41*10^5^)
	TSI>=1.8	1.62 (0.75, 2.72)	3.71 (1.03, 13.33)
	TSI<1.9	3.78 (0.88, 1.32)	1.28 (1.12, 1.49)
	TSI>=1.9	1.35 (0.38, 4.76)	3.90 (1.26, 11.94)

OR (95% CI) was calculated using piecewise regression.

### Prevalence of LOH

We thus used the criteria for the LOH identification include the combination of LOH symptom screening by the cAMS score (>=17), with lower bioavailable testosterone (cFT<210pmol/L) and decreased testosterone secretion (TSI<1.8). The overall prevalence of LOH in the study population was 5.32%, with gradual increase from 0.4% in men aged 40-44 years to 16.1% in those 75-79 years. The prevalence of LOH was also found to be increased in men with poor education, never or quit alcohol-drinking, smoking cessation, comorbidities, and living in the country area (Table 3).

**Table 3:** Prevalence and characterization of LOH.

**Variable**	**Categories**	**n**	**LOH**	**Prevalence (%)**	***P* value**
Age (years)					<0.001
	40-44	746	3	0.40	
	45-49	1047	16	1.53	
	50-54	916	14	1.53	
	55-59	1067	57	5.34	
	60-64	1046	66	6.31	
	65-69	643	75	11.66	
	70-74	335	58	17.31	
	75-79	180	29	16.11	
Residence					<0.001
	City	758	27	3.56	
	Country	5222	291	5.57	
Education					<0.001
	Illiteracy	392	45	11.48	
	Elementary school	1823	145	7.95	
	Middle school to secondary technical school	3261	115	3.53	
	Junior college and above	493	13	2.64	
Smoking					>0.05
	Never	1887	97	5.14	
	Yes	3371	178	5.28	
	Cessation	691	40	5.79	
Alcohol-drinking					<0.001
	Never	1649	111	6.73	
	Social drinker	2170	85	3.92	
	Often	1805	93	5.15	
	Quit	315	26	8.25	
Comorbidities					<0.001
	0	3341	130	3.89	
	1	1957	132	6.75	
	2	543	44	8.10	
	>=3	139	12	8.63	

*P* values were calculated by chi square (SPSS).

## DISCUSSION

Having confirmed no age-associated decline of TT in 5980 Chinese men aged 40-79 years, this study has screened these men and identified 10 symptoms that are significantly correlated with decreased levels of bioavailable testosterone (cFT) and low indices of testosterone secretion ability (TSI) using the AMS scale. We have then defined the cut-off scores for the 10 symptoms, the thresholds for cFT and TSI, respectively, for LOH diagnosis. Thus, this study is recommending the identification for LOH include the origin of testosterone decline (TSI), the presence of testosterone deficiency (cFT), and the multiple symptoms attributable to the testosterone deficiency (cAMS scores). According to the definition of LOH as well as the physiology and regulation of testosterone, the above recommendation appears to be reasonable. Our results also emphasize that, along with aging, testosterone secretion is diminished leading to the deficiency of bioavailable testosterone, which in turn causes LOH, in spite of normal serum TT levels in aging men.

According the definition of LOH, decreased testosterone secretion and testosterone deficiency form the basis of the pathogenesis for LOH. TT/LH ratio (TSI) and cFT are decreased significantly with aging, demonstrating declines of testicular testosterone secreting function and bioavailable testosterone with advancing age, which is consistent with the basic pathogenesis of LOH. Testosterone is mainly secreted from testicular Leydig cells. In aged males, the total number of Leydig cells are decreased dramatically (about 50%) resulting in impaired functions, which is the major cause of declined testicular function during aging [17, 18]. At the same time, LH level is increased with aging, indicating a compensatory response to testicular function decline. Considering the coexistence of testicular function decline and the compensatory response in aging males, it is reasonable to recommend TSI to be included in the LOH diagnosis. A lower TSI can denote a declined but not sufficiently compensated testicular function and the hypothalamic- pituitary failure, which is considered to be the basic pathogenesis of LOH [1, 3, 12]. In the present study, most (8 of 10) items correlated with cFT also have significant relationships with TSI.

Although TT is usually used in the diagnosis of LOH, its change in aging men is slight and inconsistent [2, 4–8]. Except the compensative effect of LH, the increasing SHBG levels along with aging may bind to and store more testosterone, thus attenuate the decline of TT. TT can not accurately reflect androgen status under this situation [19]. Moreover, it appears that ethnic and lifestyle factors may contribute to the inconsistent change of TT. In most studies from western countries, slight age-associated decline in TT has been observed [2, 4], however, no age-associated declines in TT have been found in studies using large sample size in Asian men [5–8]. Weight gain and smoking cessation are consistently found to be correlated with age-associated declines in TT in longitudinal studies from two independent studies [4, 20]. Body weight and weight change vary among different ethnic groups [21, 22]. In Caucasian men, >25% of men have BMI≧30 (obese) [4, 20], while in the present study, only 4.9% of men have BMI≧30 and 13.0% of men have BMI≧28 (obese in Chinese standard). Smoking cessation rate is much lower (11.6%) in the present study than that in a Caucasian population (48.8%) [2]. Similar difference in smoking cessation rates has been reported previously [23, 24]. On the other hand, only 2 items (in psychological subscale) from the AMS scale and 2 items from the SF-36 scale has been found to be correlated with TT, contrary to 10 items from the AMS and 9 items from the SF-36, respectively, correlating with cFT. Moreover, no sexual symptoms was found to be correlated with TT, but instead, 4 of them are found to be correlated with cFT. These results suggest that cFT, a form of bioavailable testosterone, may better serve as an indicator for testosterone deficiency in aging men than TT. A report also demonstrated that low cFT was associated with hypogonadal symptoms in men with normal TT levels [25].

Among the scales for screening LOH symptoms, AMS remains the most widely used [15]. The present study has identified symptoms correlated with cFT to form a concise version of AMS (cAMS). The cut-off score of the cAMS (≧17) is defined as the threshold because aging males with a score above 17 have significantly lower cFT and TSI. Interestingly, this score is close to the suggested score of AMS scale for LOH screening (score 27 for 17 items) [15]. Moreover, these 10 items show uniform distribution in the three subscales, of which 3 items from psychological subscale, 3 items from somatic subscale, and 4 items from sexual subscale, respectively [15]. Importantly, among these 10 items, 10 of 10 are correlated with cFT and 8 of 10 are correlated with TSI and therefore, they systematically reflect the symptoms caused by the testosterone deficiency and are reasonable for the LOH screening according to the definition and pathogenesis of LOH.

Using the cAMS scores in this study, the participants were classified into symptomatic and asymptomatic categories. The probability of the symptoms is increased with decreased levels of cFT, FTI, and TSI, respectively, confirming the negative correlations between the symptoms and the deficiency of bioavailable testosterone as well as the decompensated testicular functional decline. Further analysis has identified the thresholds of cFT, FTI, and TSI, respectively, below which the probability of the symptoms becomes increasingly prevalent in the training set. Similar thresholds of cFT and TSI have been identified in the validation set. The thresholds of cFT, which represents the bioavailable testosterone, and TSI, which implies the impaired testosterone secretion, and in combination with the cAMS, which includes symptoms correlated with testosterone deficiency, are thus recommended to constitute the comprehensive criteria proposed for the diagnosis of LOH.

Taken together, the present study recommended the identification for LOH include impaired testosterone secretion ability (TSI<1.8), deficiency of bioavailable testosterone (cFT<210 pmol/L) and correlated multiple hypogonadal symptoms (cAMS≧17), respectively. This criteria could be adopted or modified for LOH identification in Asians and the population without obvious TT decline during aging. On the other hand, sales of commercial testosterone products have increased substantially among older men during the past decade [26]. Many men initiate testosterone replacement therapy without testosterone testing [27, 28]. However, the testosterone replacement treatment in LOH men is under extensive debate [11, 12, 29, 30]. Our results suggest that the diminished testosterone secretion and the deficiency of bioavailable testosterone, other than the total testosterone, may better explain the cause and pathogenesis of LOH. And the criteria recommended here for LOH identification were developed based on evidence and according to the definition of LOH, with emphasize on decreased testosterone secretion and bioavailable testosterone levels. Thus our results proposed here may provide a new direction in LOH treatment emphasizing the improvement of testicular secretion of testosterone and the elevation of serum levels of bioavailable testosterone.

## MATERIALS AND METHODS

### Study design and participants

From June 1, 2013 to August 31, 2016, a multiple-center cross-sectional survey including six representative areas (provinces) of China was conducted. Multistage random, cluster sampling was performed in this study. Purposive sampling was used at the first stage: east (Jiangsu), southwest (Guizhou), northeast (Shanxi), north (Hebei), south (Guangdong), and the center (Hubei) of China were selected based on the status of economy, life style, and population distribution (Supplementary Figure 1); Stratified cluster sampling was used at the second stage: communities were stratified by urban, suburban, and rural status, communities within the three strata were random sampled with proportional allocation from each locate (province). At the third stage, all men between 22 to 79 years in the selected communities (villages) were informed to attend this study. This study was approved by the Ethical Committee Review Board of Tongji Medical College (NO. 2013S073). Written informed consent was obtained from all participants.

### Questionnaires

The participants were invited to fill interviewer-assisted questionnaires including basic information, history of disease, the simplified Chinese version of the AMS scale [15, 16], the Medical Outcomes Study 36-Item Short-Form Healthy Survey (SF-36), and the Beck Depression Inventory. All questionnaires were in simplified Chinese, and local interviewers were trained for the assistance to old participants in some areas with dialect.

### Measurements

Height, weight, circumference of abdomen and chest, and blood pressure were measured. A fasting venous blood sample for the hormone measurement was obtained from each participant in the morning between 7:00 and 11:00. Serum was processed, stored at -70° C, and assayed later for measurement of TT, LH), and SHBG by chemiluminescent immunoassays on a Beckman Access Immunoassay system (Beckman coulter, Inc., USA). Between-day imprecision (coefficient of variation) was 8.10 and 6.26% at 0.35 and 12.88 ng/ml, 5.4 and 5.2% at 6.3 and 171 nmol/L, and 6.4 and 5.4% at 4.01 and 55.04 IU/L for TT, SHBG and LH, respectively. cFT was calculated from testosterone and SHBG using mass action equations as previously described [14]. TSI and FTI was calculated using TT/LH equation and TT/SHBG equation, respectively.

### Statistical analysis

Questionnaire and hormone data were proofread and entered in EPIdata version 3.02 (Odense, Denmark). Data analyses were performed with SPSS version 18.0 (Armonk, NY, USA), and some graphics were produced using both SPSS and Stata 13.0 (College Station, TX, USA). A *P*-value <0.05 was considered statistically significant. In all subjects, age trend of hormones was analyzed using multivariate multiple regression; and the differences of hormone levels among BMI categories were compared by Games-Howell. Subjects were then randomly subdivided into a training set and a validation set. In the training set, symptoms associated with testosterone levels and secretion were screened with proportional ordinal logistic regression, and were further confirmed by Mann-Whitney U test. The confirmed symptoms were used to form the scale of LOH symptoms. In both the training set and validation set, the trend of the probability of LOH symptom along with the change of hormone levels was analyzed by Lowess smoothing, and the thresholds of cFT and FTI were identified with piecewise repression.

## Supplementary Material

Supplementary Figure 1

Supplementary Tables
