# Supplementary material for: Decreased testosterone secretion index and free testosterone level with multiple symptoms for late-onset hypogonadism identification: a nationwide multicenter study with 5980 aging males in China
Source: Aging (Albany NY). 2020 Nov 21;12(24):26012–28. doi: 10.18632/aging.202227 (PMC7803574; doi:10.18632/aging.202227)
Supplement: Supplementary Figure 1 [file aging-12-202227-s001.pdf]

SUPPLEMENTARY FIGURE

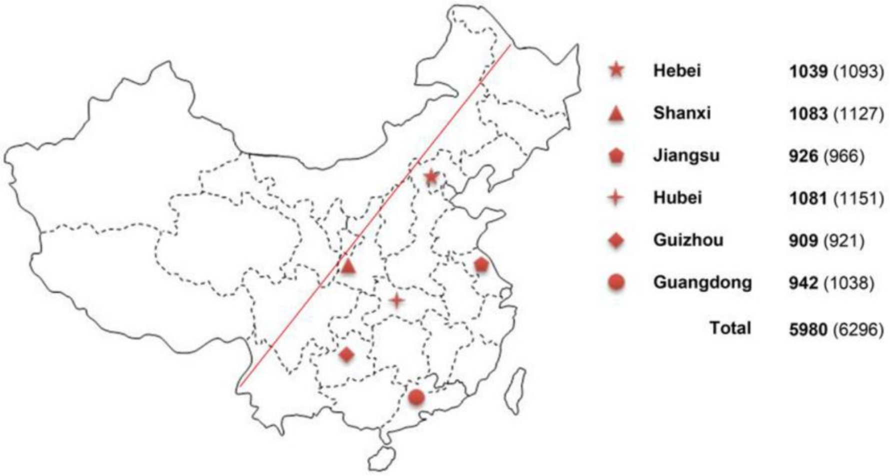

Supplementary Figure 1. Participants recruited from six representative areas (provinces) in China with relatively dense population. Population of the areas on the right of the red line accounts for 94% of total population of China. Numbers in the bracket denote the total number before the exclusion.
