# Supplementary material for: Decreased testosterone secretion index and free testosterone level with multiple symptoms for late-onset hypogonadism identification: a nationwide multicenter study with 5980 aging males in China
Source: Aging (Albany NY). 2020 Nov 21;12(24):26012–28. doi: 10.18632/aging.202227 (PMC7803574; doi:10.18632/aging.202227)
Supplement: Supplementary Tables [file aging-12-202227-s002.pdf]

SUPPLEMENTARY TABLES

Supplementary Table 1. Hormone differences among BMI groups.

Hormones	BMI group 1	BMI group 2	*P* value
TT	BMI <24	24=<BMI<28	<0.001
	BMI<24	BMI>=28	<0.001
	24=<BMI<28	BMI>=28	<0.001
cFT	BMI <24	24=<BMI<28	<0.001
	BMI<24	BMI>=28	0.124
	24=<BMI<28	BMI>=28	0.344
LH	BMI <24	24=<BMI<28	<0.001
	BMI<24	BMI>=28	<0.001
	24=<BMI<28	BMI>=28	0.335
SHBG	BMI <24	24=<BMI<28	<0.001
	BMI<24	BMI>=28	<0.001
	24=<BMI<28	BMI>=28	<0.001
FTI	BMI <24	24=<BMI<28	<0.001
	BMI<24	BMI>=28	<0.001
	24=<BMI<28	BMI>=28	<0.001
TSI	BMI <24	24=<BMI<28	0.587
	BMI<24	BMI>=28	<0.001
	24=<BMI<28	BMI>=28	<0.001

Supplementary Table 2. Factors associated with TT levels.

Factors	β	p value	β (95% CI)
BMI	-0.511	0.000	(-0.550， -0.473)
Residence	0.592	0.000	(0.440， 0.745)
Smoking	0.383	0.000	(0.180， 0.585)
Alcohol-drinking	-0.261	0.000	(-0.405， -0.117)
Marital status	0.527	0.002	(0.200， 0.854)
Age	-0.009	0.199	(-0.023， 0.005)

Factors associated with TT levels were screened using stepwise multiple linear regression in all participants. Then relationship between TT levels and these factors and age were analyzed using multiple linear regression. Age was not correlated TT.

Supplementary Table 3. Characteristics of the training set and validation set at baseline.

Variable	Training Set (n=2978)	Validation Set (n=3002)
**Age and anthropometric measures**		
Age, mean (SD), years	55.8 (9.3)	56.1 (9.4)
Height, mean (SD), cm	165.3 (6.4)	165.1 (6.3)
Weight, mean (SD), kg	66.3 (10.6)	66.3 (10.6)
Body-mass index, mean (SD), kg/m^2	24.2 (3.3)	24.3 (3.3)
Waist circumference, mean (SD), cm	86.6 (11.2)	87.1 (11.0)
**Health status**		
SF-36 score, mean (SD)		
Physical functioning	83.5 (20.6)	84.0 (20.8)
Physical role	67.0 (42.8)	70.0 (41.3)
Body pain	76.4 (17.5)	77.2 (17.2)
General health	62.7 (21.2)	62.5 (20.8)
Vitality	68.8 (15.9)	69.0 (16.4)
Social role	45.2 (13.2)	45.5 (12.8)
Emotional role	73.3 (41.3)	75.3(39.7)
Mental health	68.4 (15.1)	68.1 (15.6)
Beck Depression Inventory	2.87 (4.40)	2.92 (4.32)
**Hormone levels**		
Testosterone		
TT, mean (SD), nmol/L	16.3 (5.4)	16.0 (5.0)
cFT, ean (SD), nmol/L	0.28 (0.97)	0.27 (0.86)
SHBG, mean (SD), nmol/L	46.9 (22.8)	47.0 (23.1)
**Lifestyle variables**		
Sexual-partner status, No./total No. (%)		
Partner	2926/2972(98.5)	2955/2999(98.5)
No partner	46/2972(1.5)	44/2999(1.5)
Smoking status, No./total No. (%)		
Never smoked	954/2961(32.2)	934/2990(31.2)
Former smoker	358/2961(12.1)	333/2990(11.1)
Current smoker	1649/2961(55.7)	1721/2990(57.6)
Alcohol intake, No./total No. (%)		
None	848/2953(28.7)	801/2986(26.8)
Occasionally	1058/2953(35.8)	1112/2986(37.2)
Often	887/2953(30.1)	918/2986(30.8)
Stop drinking	160/2953(5.4)	155/2986(5.2)
Body-mass index, No./total No. (%)		
<25	1815/2978(61.0)	1800/3002(59.8)
≥25 to <30	1021/2978(34.3)	1053/3002(35.1)
≥30	142/2978(4.8)	149/3002(5.0)
Coexisting illness, No./total No. (%)		
Number		
0	2008/2978(67.4)	1927/3002(64.2)
1	715/2978(24.0)	811/3002(27.0)
≥2	257/2978(8.6)	265/3002(8.8)
Type		
Heart disease	128/2978(4.3)	131/3002(4.4)
Hypertension	508/2978(17.0)	586/3002(19.5)
Chronic bronchitis or asthma	208/2978(7.0)	215/3002(7.2)
Diabetes	111/2978(3.7)	130/3002(4.3)

Prostate disease	141/2978(4.7)	139/3002(4.6)
Cancer	19/2878(0.6)	23/3002(0.8)
Stroke	76/2978(2.6)	73/3002(2.4)
Tuberculosis	39/2978(1.3)	41/3002(1.4)
Hepatitis	59/2978(2.0)	65/3002(2.2)

Supplementary Table 4. Symptoms screening by ordinal regression.

Scale	Symptoms	*P* value
AMS scale		
	Decline in your feeling of general well-being	0.087
	Joint pain and muscular ache	0.112
	Excessive sweating	0.020
	Sleep problems	0.051
	Increased need for sleep, often feeling tired	0.000
	Irritability	0.000
	Nervousness	0.001
	Anxiety	0.003
	Physical exhaustion / lacking vitality	0.001
	Decrease in muscular strength	0.000
	Depressive mood	0.208
	Feeling that you have passed your peak	0.764
	Feeling burnt out, having hit rock-bottom	0.001
	Decrease in beard growth	0.000
	Decrease in ability/frequency to perform sexually	0.000
	Decrease in the number of morning erections	0.000
	Decrease in sexual desire/libido	0.000
SF-36		
	In general, would you say your health is ?	0.531
	Compared to one year ago, how would your rate your health in general now?	0.000
	Does your health now limit you in:	
	Vigorous activities	0.009
	Moderate activities	0.000
	Lifting or carrying groceries	0.261
	Climbing several flights of stairs	0.000
	Climbing one flight of stairs	0.001
	Bending, kneeling, or stooping	0.074
	Walking more than 1500 meters	0.027
	Walking 800 meters	0.004
	Walking 100 meters	0.031
	Bathing or dressing yourself	0.060
	During the past 4 weeks, have you had any of the following problems with your work or other regular daily activities as a result of your physical health?	
	Cut down the amount of time you spent on work or other activities	0.000
	Accomplished less than you would like	0.000
	Were limited in the kind of work or other activities	0.000
	Had difficulty performing the work or other activities	0.000
	During the past 4 weeks, have you had any of the following problems with your work or other regular daily activities as a result of any emotional problems ?	
	Cut down the amount of time you spent on work or other activities	0.191
	Accomplished less than you would like	0.004
	Didn't do work or other activities as carefully as usual	0.008
	During the past 4 weeks, to what extent has your physical health or emotional problems interfered with your normal social activities with family, friends, neighbors, or groups?	0.040

	How much bodily pain have you had during the past 4 weeks?	0.148
	During the past 4 weeks, how much did pain interfere with your normal work?	0.012
	These questions are about how you feel and how things have been with you during the past 4 weeks. For each question, please give the one answer that comes closest to the way you have been feeling.	
	Did you feel full of pep?	0.11
	Have you been a very nervous person?	0.000
	Have you felt so down in the dumps that nothing could cheer you up?	0.012
	Have you felt calm and peaceful?	0.519
	Did you have a lot of energy?	0.000
	Have you felt downhearted and blue?	0.106
	Did you feel worn out?	0.144
	Have you been a happy person?	0.572
	Did you feel tired?	0.369
	During the past 4 weeks, how much of the time has your physical health or emotional problems interfered with your social activities?	0.001
	I seem to get sick a little easier than other people	0.149
	I am as healthy as anybody I know	0.094
	I expect my health to get worse	0.233
	My health is excellent	0.022
Beck Depression Inventory		
	Sadness	0.008
	Pessimism	0.133
	Past failure	0.001
	Loss of pleasure	0.044
	Guilty feeling	0.005
	Self-dislike	0.228
	Suicidal thoughts	0.082
	Lost of interest	0.001
	Indecisiveness	0.051
	Worthlessness	0.017
	Loss of energy	0.000
	Tiredness or fatigue	0.677
	Changes in appetite	0.182

Supplementary Table 5. Differences in hormones between subjects divided by cAMS scores.

cAMS Score	*P* value			
	TT	cFT	TSI	FTI
11	0.673	0.363	0.481	0.416
12	0.884	0.209	0.339	0.272
13	0.949	0.308	0.184	0.610
14	0.694	0.231	0.039	0.277
15	0.975	0.130	0.047	0.054
16	0.905	0.079	0.024	0.012
17	0.491	0.003	0.010	0.006
18	0.926	0.005	0.069	0.008
19	0.382	0.005	0.079	0.008
20	0.373	0.004	0.044	0.005

P values were calculated by student t test.
